# Will cellular immunotherapies end neurodegenerative diseases?

**DOI:** 10.1016/j.it.2024.03.006

**Published:** 2024-04-09

**Authors:** Pavle Boskovic, Wenqing Gao, Jonathan Kipnis

**Affiliations:** 1 Brain Immunology and Glia (BIG) Center, Washington University in St Louis, St Louis, MO 63110, USA; 2 Department of Pathology and Immunology, School of Medicine, Washington University in St Louis, St Louis, MO 63110, USA

## Abstract

Neurodegenerative disorders present major challenges to global health, exacerbated by an aging population and the absence of therapies. Despite diverse pathological manifestations, they share a common hallmark, loosely termed ‘neuroinflammation’. The prevailing dogma is that the immune system is an active contributor to neurodegeneration; however, recent evidence challenges this. By analogy with road construction, which causes temporary closures and disruptions, the immune system’s actions in the central nervous system (CNS) might initially appear destructive, and might even cause harm, while aiming to combat neurodegeneration. We propose that the application of cellular immunotherapies to coordinate the immune response towards remodeling might pave the way for new modes of tackling the roadblocks of neurodegenerative diseases.

## CNS immune privilege: an updated concept

The concept of CNS immune privilege, once thought to isolate the mammalian brain from systemic immunity, has evolved, with research demonstrating the beneficial roles of immune cells in CNS health and disease, as well as the existence of physical routes of communication between the two systems [1,2]. Even though microglia, the innate myeloid cells of the CNS, are the major immune cell type within the CNS parenchyma in homeostasis, adaptive immune cells play crucial roles in regulating brain function [3,4]. These effects are likely mediated through secretion of cytokines and other mediators [5–8]. Albeit surprising if viewed through the lens of the narrowly defined ‘CNS immune privilege’, these findings resonate well with recent discoveries of close communication between the immune system and the brain [2,9–12].

**Figure 1.**
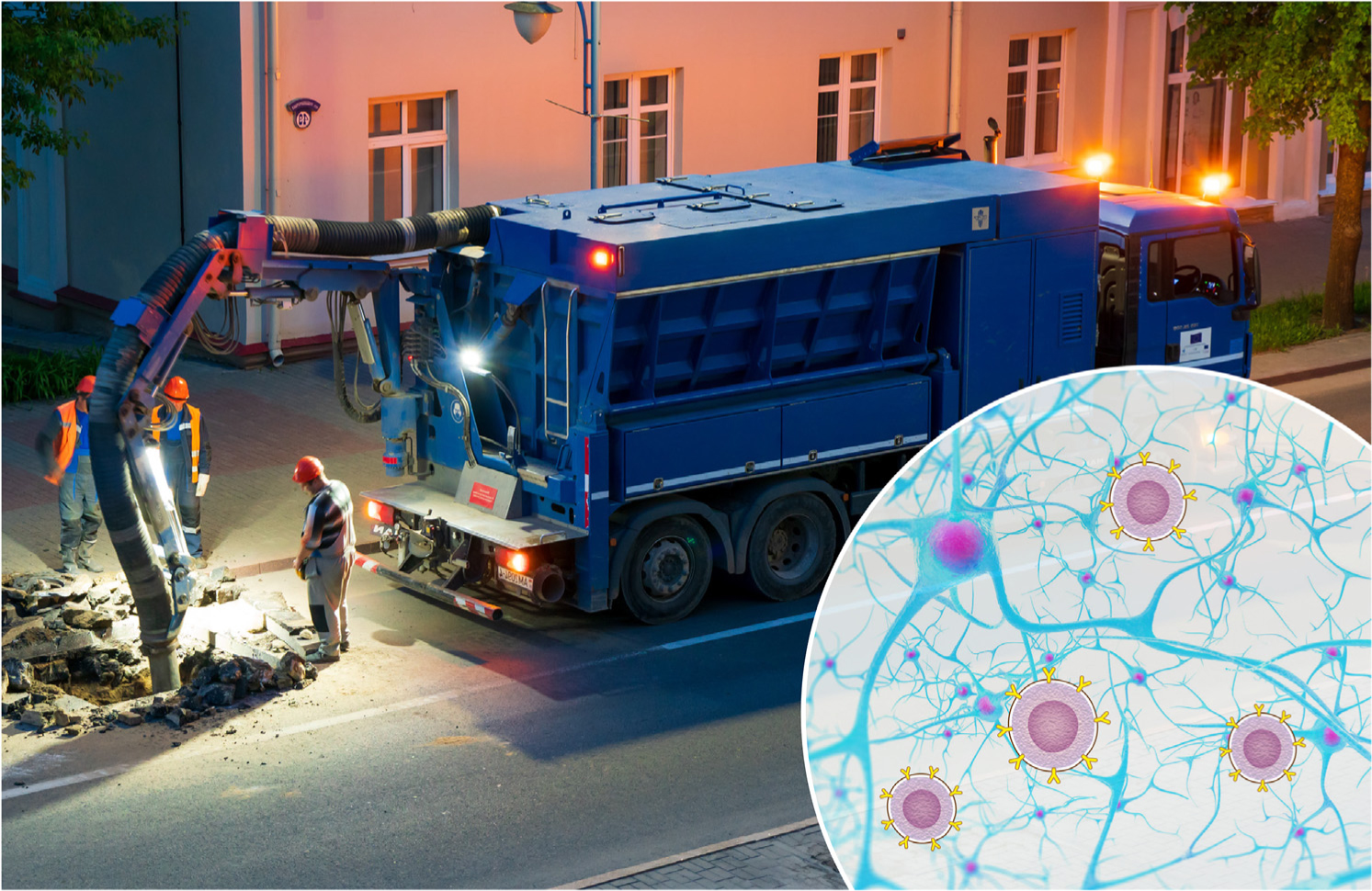
This figure exemplifies our analogy of the role of the immune system in neurodegenerative disorders being akin to that of road construction. The inconvenience of road construction, as shown in the top panel, creates roadblocks, traffic jams, and short- to long-term reroutes that inconvenience commuters and disrupt the network in its entirety. Similarly, immune cells undergo efforts to mediate neurodegenerative diseases and central nervous system (CNS) injuries, seemingly exacerbating the pathology but with the ultimate goal of resolving the underlying cause. Introduction of modern equipment and coordinated management, as seen in the lower panel, focuses working efforts and ensures a smooth transition towards the completion of reconstruction tasks. Analogously, we propose that the introduction of immune cellular therapy – aimed at coordinating the existing immune response and resolving the root cause of neurodegenerative diseases – might substantially alter the course of the disease and mitigate pathology.

The interplay between immune cells and the CNS resembles an elaborate network of roads in a crowded city. While roadwork is temporarily inconvenient, resulting in reroutes and delays, and may frustrate commuters, it is essential for maintaining the integrity of the infrastructure (Figure 1, Key figure). Analogously, immune cells act as custodians of CNS infrastructure, ensuring its maintenance, repair, and remodeling. Within the context of neurodegenerative diseases, immune activity can create roadblocks that, if not resolved, contribute to system deterioration. However, the complete elimination of these cells does not address the underlying problem. Although in animal models such cellular elimination may result in acute improvements, it is akin to how a traffic jam might be acutely resolved if a construction crew were eliminated from the site even before the problem were fixed.

In this opinion article we discuss the current understanding of the role of the adaptive immune system in shaping neurodegenerative diseases and outline the seemingly contradictory evidence published thus far regarding the role of immunity in the degenerating CNS. We propose cellular immunotherapy as a possible new avenue towards treating neurodegenerative diseases. Similar to how the introduction of updated tools along with efficient management of an otherwise stalled construction site can contribute to the resolution of roadblocks and normalization of the network, we posit that introducing cells capable of coordinating the immune milieu in neurodegenerative diseases can contribute towards the resolution of the underlying pathology.

## Key figure

### The immune system: the body’s construction crew

**Figure:**
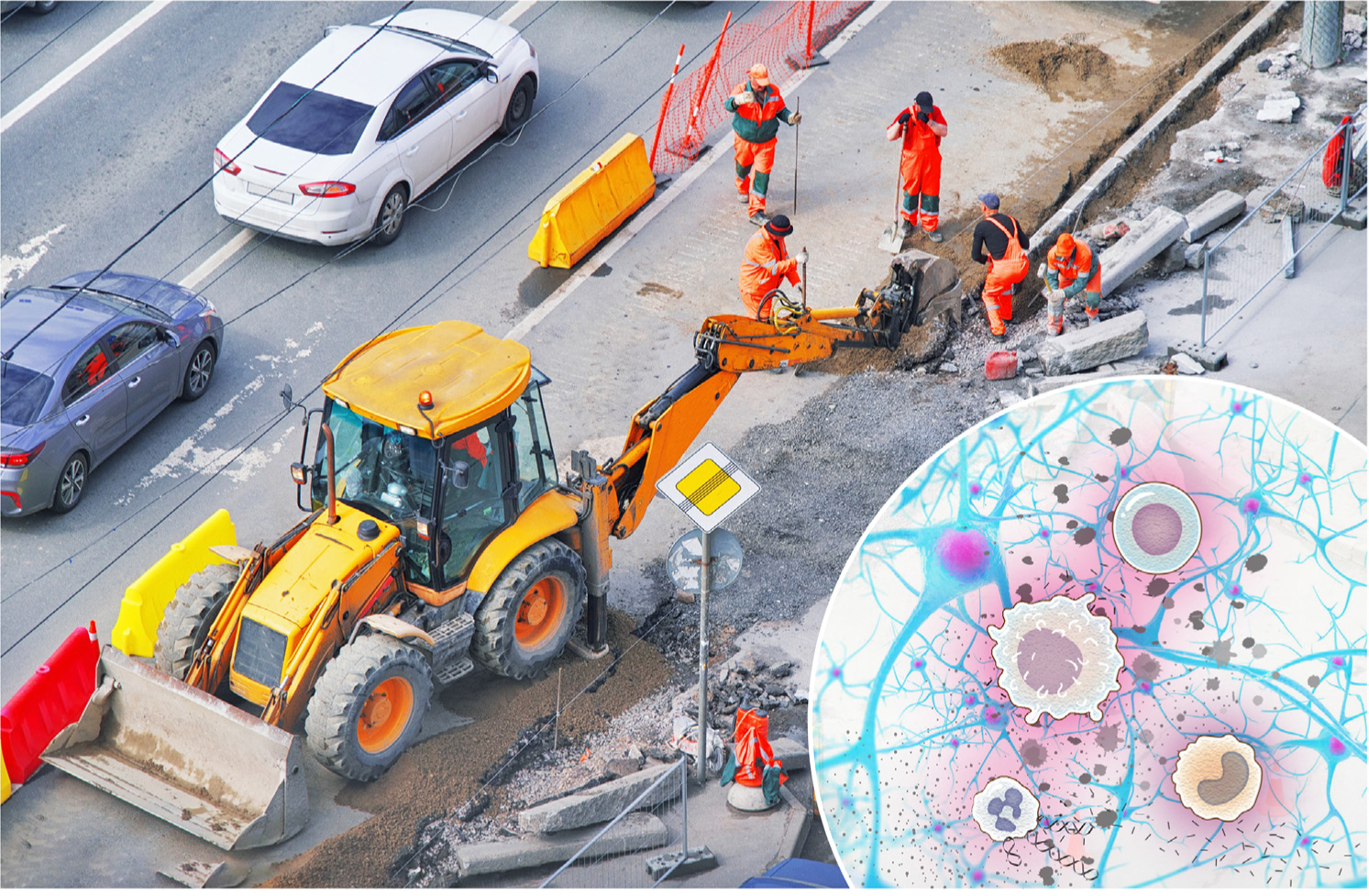


## The ambiguous role of immune cells in neurodegenerative diseases

The impact of immune cells in neurodegenerative diseases is a complex topic, often under contention. Nevertheless, through myriad published articles with evidently opposite conclusions, what emerges is that neurodegenerative disorders (chronic and acute) are invariably associated with the involvement of innate [13–15] and adaptive [16–22] immunity.

Microglia, the resident myeloid population of the CNS, mediate innate immune responses while maintaining CNS homeostasis [23,24]. Indeed, advances in single-cell sequencing technology are enabling in-depth characterization of microglial states in homeostasis, throughout aging, in CNS injury, and in various neurodegenerative diseases in mice and humans [13,25]. In CNS neurodegenerative conditions, microglia adopt a **damage-associated microglia (DAM)** (see Glossary) phenotype characterized by the expression of genes such as Apoe, *Trem2, Itgax*, and others [14,26]. Studies on TREM2, a receptor thought to contribute to the DAM phenotype, have shown opposing outcomes where its depletion results in improved or worsened disease in **PS19** (**tauopathy**) and **5xFAD** (**amyloidosis**) Alzheimer’s disease (AD) mouse models, respectively [27,28]. In the PS19 model, deletion of TREM2 resulted in significantly less brain atrophy and ameliorated the microgliosis phenotype [27]; by contrast, in the 5xFAD model, TREM2 deficiency exacerbated amyloidosis and neuronal loss [28]. Similarly, the complete depletion of microglia has yielded divergent outcomes in different models of AD, further emphasizing the nuanced role of these cells [29–31]. On the one hand, microglial cell depletion in 5xFAD mice via long-term treatment with the CSF1R inhibitor PLX5622 showed a marked reduction in amyloidosis [29], as well as tau propagation in two models of tauopathy, relative to controls [30]. On the other hand, depleting microglia resulted in exacerbated amyloid-β-dependent tau propagation in a tau-seeding mouse model [31]. These findings suggest that the role of microglial cells in AD varies based on the model used, and further research is required to understand their contribution to disease pathophysiology.

The role of T cells in neurodegenerative diseases is also a debated topic. Early studies demonstrated that T cell deficiency worsens cognition and memory [32,33]. Self-reactive T cells have been shown to aid CNS repair in animal models of optic nerve and spinal cord injury [34,35]. However, the diversity of T cell subtypes and their responses necessitates a stratified approach for translatability to human patients. In mouse models of traumatic brain injury, CD8^+^ T cells and proinflammatory CD4^+^ T cells have been detrimental towards recovery, while immunoregulatory **T helper 2 (Th2) cells** can promote injury repair and neuroplasticity [36–38].

In AD – based on experimental factors such as the models used, timing, and observed cellular subsets – divergent results have also been reported. For example, complete depletion of adaptive immunity through a knockout of the *Rag2* gene showed either beneficial or detrimental effects in 5xFAD and APP/PS1 models of amyloidosis that were linked to amyloid plaque load and microglial activation [19,20]. However, amyloid β-specific **T helper 1 (Th1) cells** provided benefit in 5xFAD and APP models of AD by enhancing plaque clearance and improving cognition, with conflicting results regarding Th2 cells [39,40]. In line with effector CD4^+^ T cells being reported as beneficial against AD, other studies have shown a negative effect of CD4^+^ **regulatory T cells (Tregs)** in AD, with their depletion in mice resulting in reduced amyloid load as well as improved learning and memory [16]. Conversely, T cell expansion and CNS infiltration in a tau mouse model of AD was shown to be detrimental, exacerbating neurodegeneration [17]. In both amyloidosis and tauopathy mouse models, however, immune checkpoint therapy against inhibitory receptor programmed death 1 (PD-1) showed promise in controlling pathology through different proposed mechanisms [17,41]. While it was suggested that anti-PD-1 immunotherapy acted to increase the Treg population in the tauopathy model [17], in the amyloid models [41] anti-PD-1 antibody therapy was proposed to induce a systemic interferon γ (IFNγ) response. Moreover, a study using human cerebrospinal fluid (CSF) and brain tissue showed a strong negative correlation between the presence of CD8^+^ effector memory T cells and cognition in AD and mild cognitive impairment (MCI) patients [22].

In summary, deciphering the role of the immune system in neurodegenerative diseases is fraught with complexity, and a consensus on the differential roles of adaptive and innate immune cells remains to be reached: a problem which has persisted for a long time. However, it is evident that both innate and adaptive immunity play pivotal roles, and indiscriminate modulation of immune populations may yield unpredictable outcomes. Thus, we advocate for a tailored approach that utilizes cell-based immunotherapies to reshape the immune milieu towards a constructive phenotype while preserving its essential components.

## Cellular immunotherapy in CNS diseases and injuries: mitigating roadblocks

The groundwork for applying cell-based immunotherapies to treat neurodegenerative diseases and CNS injuries was laid long ago [4,34,35,38,39]. As discussed, the utilization of CNS antigen-specific T cells significantly improved recovery following optic nerve crush and spinal cord injury in murine models [34,35]. Similarly, Treg cells engineered to express myelin basic protein (MBP)- or myelin oligodendrocyte glycoprotein (MOG)-specific **chimeric antigen receptor (CAR)-T cells** demonstrated their potential by alleviating symptoms of experimental autoimmune encephalomyelitis (EAE) (mouse model of multiple sclerosis, MS) [42]. Although more studies are warranted, this body of research holds promise for leveraging T cell-based therapies in combating neurodegenerative diseases, not by removing specific cell populations but rather by reshaping the immune microenvironment associated with diseased sites.

The translatability of these studies presents a major challenge because the active immunization of humans to raise antigen-specific T cells [43–45] is not feasible, due to risk of autoimmune disease development. Fortunately, advancements in single-cell sequencing [46] and protein engineering [47] offer potential new solutions, bringing us closer to the era of personalized cellular immunotherapeutics for CNS injuries and neurodegenerative disorders. Strategies such as T cell receptor (TCR)-transgenic autologous T cells or off-the-shelf approaches such as those provided by CAR-T cell therapy might help to streamline the production of candidate cell-based immunotherapies.

## TCR-transgenic autologous T cells

The era of single-cell sequencing has seen tremendous advances in our understanding of cellular diversity in health and disease [13,46]. Single-cell V(D)J sequencing provides detailed information on T cell clonality and the exact sequence of TCR for each cell, enabling characterization of the T cell repertoire at an unprecedented level of detail. The accessibility of these techniques offers hope that the development of neuroprotective TCR-transgenic autologous T cells could be achieved in the foreseeable future.

**Figure 2.**
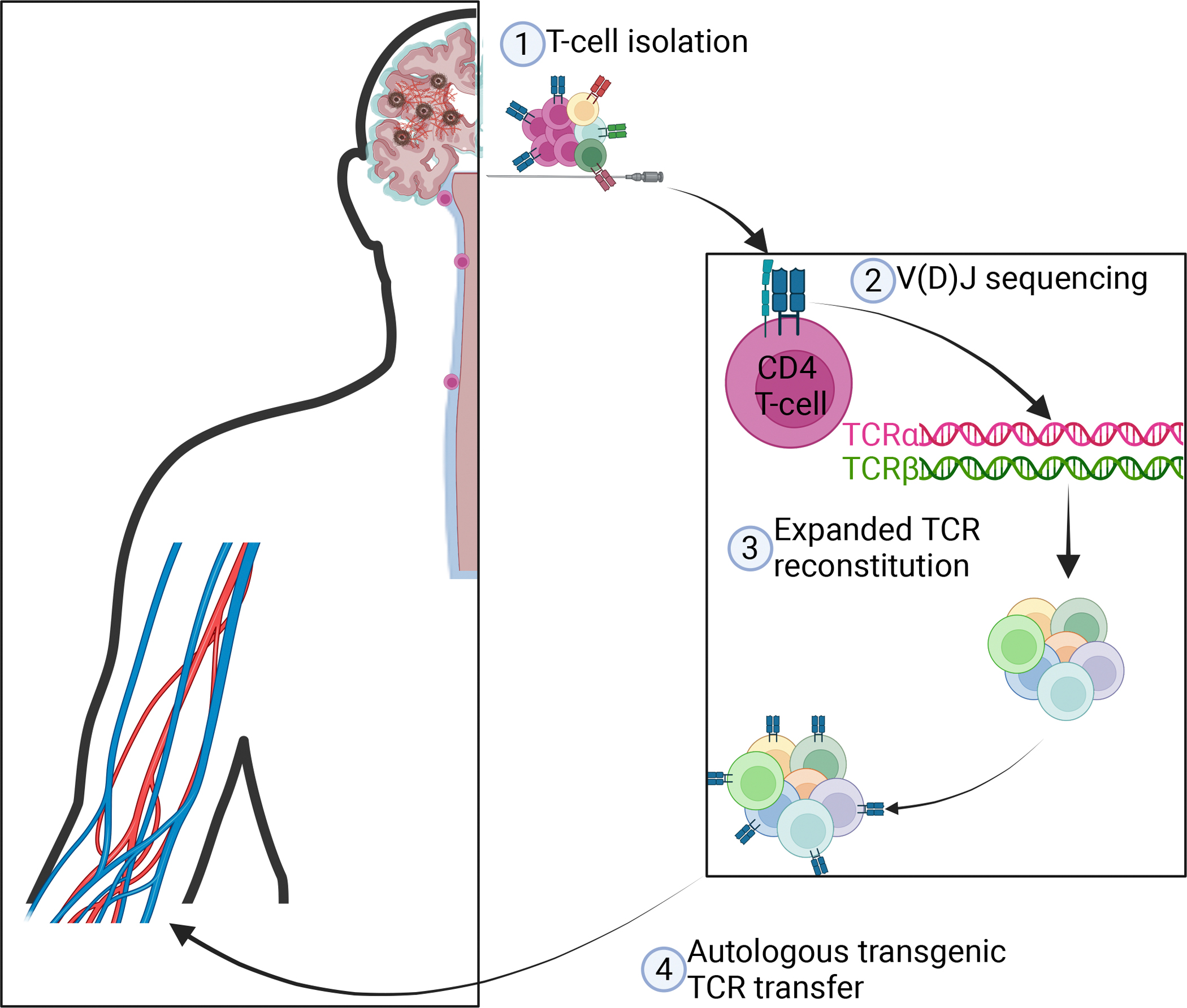
Model for T cell receptor (TCR) reconstitution and autologous TCR-transgenic T cell therapy. Patients presenting with neurodegenerative disorders such as Alzheimer’s disease (AD) could potentially benefit from a personalized immunotherapy approach. T cells can be identified in the cerebrospinal fluid (CSF) of these patients ① and submitted to single-cell V(D)J sequencing to identify expanded clones ②. Once identified, TCR molecules belonging to expanded T cell clones are reconstituted on the autologous T cells of the patient ③ and reintroduced into the patient ④. Figure created with Biorender.com.

When designing T cell-based therapy, several criteria must be considered. Discovering TCRs for a specific antigen without prior immunization is a major challenge. Instead, by utilizing single-cell TCR sequencing, naturally occurring expanded T cell clones that are responsive to relevant antigens can be identified, and the properties of their TCRs assessed *in vitro* to select the ones that are most suitable for therapeutic application. Despite tissue-resident CD8^+^ T cells showing beneficial effects in mouse models of AD [21], an unwarranted CD8^+^ T cell response should be avoided as it could potentially cause cytotoxic effects. By restricting the sequencing-based discovery approach to expanded CD4^+^ T cell clones, we can ensure that the respective TCRs respond specifically to antigens presented within the context of the major histocompatibility complex (MHC) class II molecules. Finally, having shown the most promise in neurodegenerative diseases, introducing these TCR molecules to a pre-stratified autologous CD4^+^ T cell population [32,38,39] could ensure both minimal cytotoxicity and a lack of rejection or induction of graft-versus-host disease (Figure 2).

We propose that applying these criteria to a personalized medicine approach for patients suffering from neurodegenerative diseases such as AD or amyotrophic lateral sclerosis (ALS) may help to elicit a protective T cell response that supports neuronal health and mitigates degenerative processes, a possibility that merits further attention.

## Off-the-shelf neuroprotective T cells

Major recent advancements in cancer therapy have been achieved through the application of CAR-T cell technology [48]. CAR molecules are cell-surface chimeric receptors consisting of a single-chain antibody with known specificity coupled with various combinations of transmembrane and costimulatory domains, and a CD3 signaling domain. Upon antigen recognition, these molecules activate downstream TCR signaling pathways [47]. CAR-equipped CD8^+^ T cells targeting tumor-specific antigens or neoantigens have thus far shown great potential in combating certain hematological malignancies by targeting cell-surface proteins that are ubiquitously expressed on tumor cells, such as CD19 [49,50].

**Figure 3.**
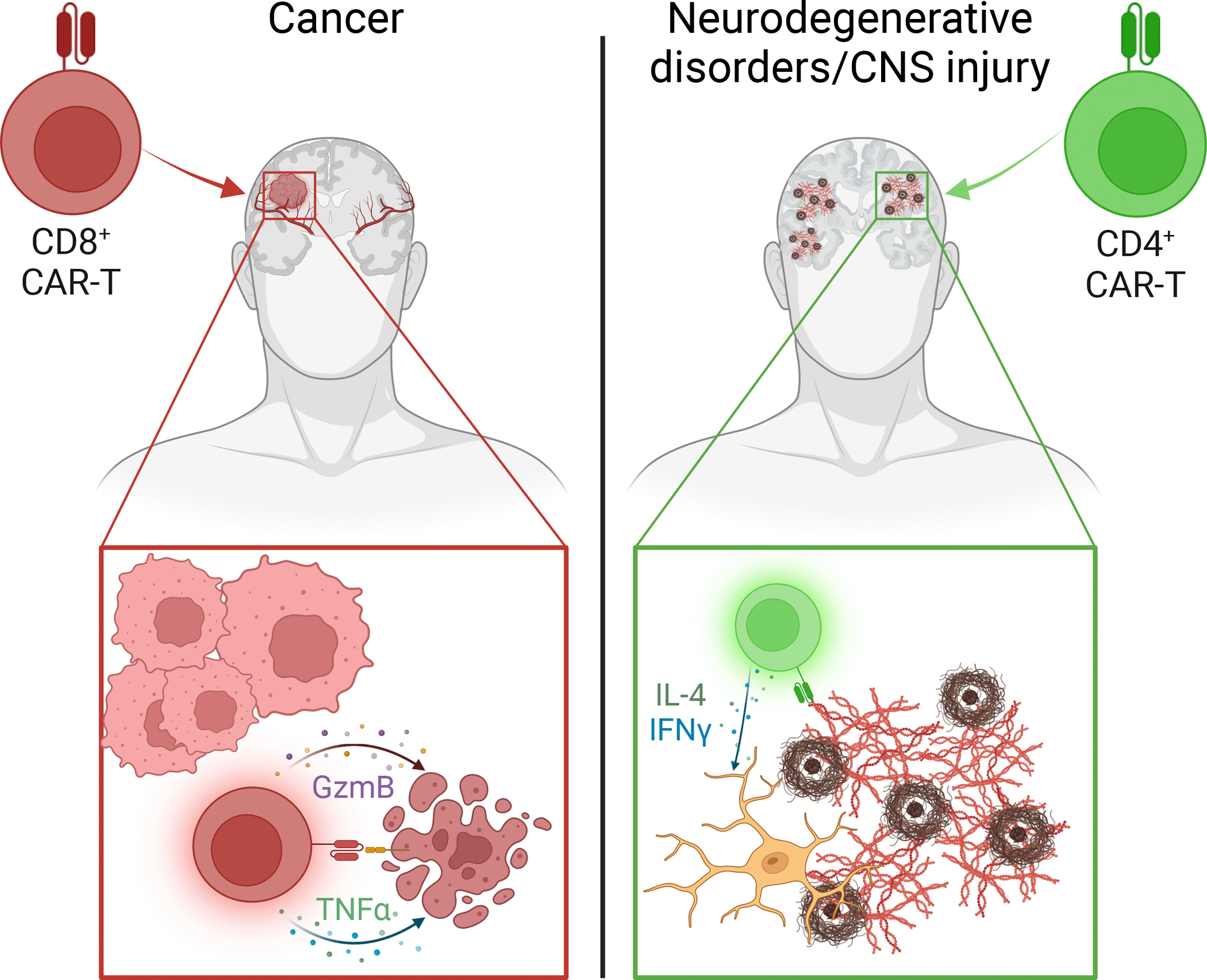
Model for adapting chimeric antigen receptor (CAR)-T cell immunotherapy in neurodegenerative diseases. By equipping CD8^+^ T cells with CAR molecules targeting tumor-specific surface antigens or **neoepitopes**, one can target the cytotoxic action of these cells towards killing tumor cells through the secretion of granzymes, perforins, and cytokines (left). As a similar yet alternative approach, we propose that to treat certain neurodegenerative diseases, such as Alzheimer’s disease (AD) or central nervous system (CNS) injury, CD4^+^ T cells (instead of CD8^+^ T cells) might be equipped with CAR molecules, targeting pathology-specific antigens. CD4^+^ CAR-T cells would be expected to act beneficially by mediating neuroinflammation through the secretion of cytokines known to provide certain benefits and coordinating the innate immune response (i.e., myeloid cell populations such as microglia) (right). Abbreviations: GzmB, granzyme B; IFNγ, interferon γ; IL-4, interleukin 4; TNFα, tumor necrosis factor α. Figure created with Biorender.

While the direct application of this technology to neurodegenerative diseases might not be feasible, due in part to the cytotoxic potential of CD8^+^ T cells, its broader potential is noteworthy. For instance, recent studies have demonstrated the utility of CAR-T cells in combating certain inflammatory and autoimmune conditions, such as viral infection and EAE, by expressing the CAR molecules on Treg cells and suppressing the otherwise detrimental immune responses [42,51]. Moreover, these results are expanding the scope of CAR technology beyond CD8^+^ T cells to include investigations into other cell types such as natural killer (NK) cells and macrophages [52,53]. Major challenges remain for the applicability of these immunotherapies to neurodegenerative diseases: namely, the identification of CNS- and pathology-specific antigens, among others. Nevertheless, extending the use of CAR molecules to CD4^+^ T cells designed to promote CNS repair could represent a substantial breakthrough in cellular immunotherapy for neurodegenerative diseases (Figure 3).

## Concluding remarks

Expanding upon the analogy between the immune system’s role in neurodegenerative diseases and the efforts of road construction crews highlights a nuanced understanding of the intricate processes underlying brain health and disease management. Just as road construction workers are equipped with an array of tools and strategies to address the diverse challenges of road maintenance, the immune system employs a complex arsenal of cells and molecules tailored to combat various threats to brain integrity. Different neurodegenerative diseases will likely require similar immunological approaches with tailored modifications for each condition, just like the construction of different roads would require specified equipment and tools, although some may be similar. Moreover, individual patients may have unique treatment needs that can be addressed by a personalized medicine approach, much as individual construction projects may require engineering solutions tailored to their specific needs.

The parallel also extends to the challenges and unintended consequences that can arise. In the same way that extensive roadwork can lead to traffic congestion, detours, and increased pollution from idling vehicles, the immune system’s response to neurodegenerative diseases can exacerbate the problem acutely, despite having the goal of overall long-term benefit. Inflammation, a hallmark of the immune response, can lead to further neuronal damage if not properly regulated [36], mirroring how poorly planned construction efforts can worsen the very issues they aim to solve.

The introduction of cellular immunotherapies into this scenario is akin to bringing in next-generation construction technologies. Just as these innovations promise to make road construction more efficient, precise, and less disruptive to the public, cellular immunotherapies might offer the hope of targeting the underlying causes of neurodegenerative diseases with specificity and effectiveness. By engineering immune cells to better recognize and eliminate the pathological features of conditions such as AD and Parkinson’s disease, these therapies might help reduce the ‘collateral damage’ seen with more generalized approaches that either inhibit or elicit certain immune responses.

Furthermore, the adaptive nature of cellular immunotherapies might enable a personalized approach to treatment. This bespoke approach ensures that the therapeutic ‘construction crew’ is not only efficient but also well integrated within the brain’s existing structures and functions, paving the way for a future where neurodegenerative diseases are managed with precision and foresight (see Outstanding questions).

The individual technologies to support the design of such therapies and their potential benefits are already established. Single-cell sequencing and the design of CAR molecules can be exploited towards discovery-based design of TCR transgenic T cells and pro-cognitive CAR-T cells. By synthesizing these methods with our understanding of the beneficial roles of T cell subpopulations in neurodegenerative diseases, we argue that it is possible to design a personalized cellular immunotherapeutic approach for chronic neurodegenerative diseases, as well as a readily available one for acute pathologies such as CNS injury.

## Highlights

‘Neuroinflammation’, one of the hallmarks of many neurodegenerative diseases, causes inadvertent damage to neuronal tissue due to the immune system’s response to the underlying pathology.

The role of the immune system in the development and progression of many neurodegenerative diseases remains elusive, with conflicting evidence in similar model systems.

Due to the complexity of the immune system’s involvement and the limitations of preclinical models, indiscriminate targeting of any single immune cell population could lead to inadvertent effects in neurodegenerative disease.

The concept of cellular immunotherapy and personalized immunotherapeutic approaches is becoming feasible with current technological advancements, and might provide a new avenue for treating neurodegenerative diseases.

## Significance

We draw a compelling analogy between the immune system’s role in neurodegeneration and a construction crew, underscoring the potential of cellular immunotherapies (especially T cell-based) to remodel and repair the central nervous system (CNS). We advocate for the development of personalized immunotherapies that leverage advanced technologies such as single-cell V(D)J sequencing and chimeric antigen receptor T (CAR-T) cell therapy. These approaches could transform the immune system from an inadvertent enabler of neurodegeneration to a precise tool for restoring the CNS: a novel strategy to combat neurodegenerative diseases.

## Outstanding questions

Can advancements in single-cell and V(D)J sequencing result in a personalized T cell therapy approach? Even though we can now identify highly expanded T cell clones responsive to self-antigens directly from the CSF of patients, whether this workflow is feasible using animal models of neurodegeneration and CNS injury remains to be demonstrated.

Is there a necessity for personalized CNS immunotherapy, or could an ‘off the-shelf’ product be developed? The time-limiting nature of conditions such as CNS injury begs the question of whether an ‘off-the-shelf’ approach is feasible for these applications, or beyond, in neurodegenerative disorders in general. This approach would likely entail the use of CAR molecules which are inherently limited to accessible antigens, an issue easily addressable in diseases presenting with extracellular protein plaque such as AD, but more complex in cases such as CNS injury.

Will immune cells be capable of repairing a damaged CNS or primarily postpone the onset of neurodegenerative diseases? Neurodegeneration involves apparently irreversible damage to the CNS. Yet it is intriguing to consider whether the continuous presence of therapeutic cells might slow down disease progression, but also enhance impaired cognition associated with these conditions. This possibility remains to be tested in preclinical models.

Would similar immune approaches be applicable for mental disorders? By modulating adaptive immunity (namely CD4^+^ T cell populations), certain results have shown benefit in psychiatric conditions such as depression and post-traumatic stress disorder in animal models. Yet given the absence of antigen specificity, it is unclear whether this strategy is broadly applicable. Should the approach prove effective in addressing previously mentioned concerns, personalized immunotherapy could offer a solution to this question.

## Glossary

Amyloidosis mouse models (5xFAD, APP/PS1): mouse models that recapitulate the amyloid-dependent pathology of the human disease, such as the formation of amyloid plaques and neuroinflammation. In the 5xFAD model, human APP and PSEN1 genes containing a total of five mutations associated with familial AD are overexpressed under the Thy1 promoter. In the APP/PS1 model, chimeric human–mouse APP and the human PSEN1 genes carrying mutations associated with familial AD are overexpressed under the Prnp promoter
Chimeric antigen receptor (CAR)-T cells: CARs are engineered cell-surface receptors consisting of a single-chain antibody domain on the surface (scFv) connected to an intracellular signaling domain. The signaling region consists of co-stimulatory domains originating from, for example, CD28 and/or CD137 (4-IBB) fused to a signaling domain of CD3 zeta (CD247). Upon recognizing the antigen of the scFv domain, these molecules promote downstream TCR signaling. CAR-T therapy is currently applied primarily in cases of hematological malignancies in which CD19-specific scFv CARs are expressed on autologous cytotoxic CD8^+^ T cells
Damage-associated microglia (DAM): a subset of microglial cells that arise in neurodegenerative diseases, aging, and CNS injury. They are characterized by distinct transcriptional profiles (increased expression of marker genes such as *Trem2, Apoe, Itgax,* and others) and altered cellular morphology
Neoepitopes: potential T/B cell antigens that arise through somatic mutations of protein-coding genes. They commonly occur in tumors with heavy mutational burdens (melanoma, types of lung cancer) or as a result of gene translocations; they could be exploited for immunotherapies targeting these tumor entities
Regulatory T cells (Tregs): a subclass of CD4^+^ T cells with immunosuppressive functions; they are crucial for maintaining tolerance against self-antigens
Tauopathy mouse models (PS19): mouse models that recapitulate the tau-dependent aspects of human disease, such as neurodegeneration, via overexpression of the human mutated microtubule-associated protein tau (*MAPT*) under the mouse prion protein (*Prnp*) promoter
T helper 1 (Th1) cells: a subpopulation of CD4^+^ T cells which act mainly in a proinflammatory fashion, stimulating the cytotoxic killing of infected cells through the secretion of cytokines such as IFNγ
T helper 2 (Th2) cells: a subpopulation of CD4^+^ T cells which, during infection, support pathogen clearance through cytokine secretion and stimulation of humoral immunity, but do not generally have cytotoxic functions
